# Perceived Stress, Depression, and Associated Factors among Undergraduate Health Science Students at Arsi University in 2019 in Oromia, Ethiopia

**DOI:** 10.1155/2020/4956234

**Published:** 2020-05-28

**Authors:** Deressa Worku, Abdisa Boka Dirriba, Berhanu Wordofa, Getahun Fetensa

**Affiliations:** ^1^Department of Nursing, College of Health Sciences, Arsi University, Oromia, Ethiopia; ^2^Department of Nursing and Midwifery, College of Health Sciences Addis Ababa University, Addis Ababa, Ethiopia; ^3^Department of Nursing, Institute of Health Science, Wollega University, Nekemte, Ethiopia

## Abstract

**Background:**

A mental health problem is a significant cause of overall disease burden globally. Among this problem, stress and depression are the central problems observed among university students due to the transitional nature. Consequently, the problem has an adverse effect on the wellbeing and academic performance of students.

**Objective:**

To assess perceived stress, depression, and associated factors among undergraduate health science students at Arsi University in 2019 in Oromia, Ethiopia.

**Methods:**

An institutional-based cross-sectional study design on undergraduate health science students was employed for the study from February 25 to April 15, 2019. Data were collected by using a self-administered questionnaire of the perceived stress scale (PSS-14) item and Beck depression inventory 21 items.

**Result:**

All of the study subjects were between ages 18 and 30 with a mean age of 20.9 ± 1.83 standard deviation years. The current prevalence of perceived stress among study subjects was 63.5%. Age category, study year, pressure to maintain a good grade, practical attachment, lack of dormitory safety, and the financial problem were identified as significantly associated factors of perceived stress. In addition to this study, results also revealed that the prevalence of depression among students was 4.4% in which thinking about career future prospects was a significantly associated factor.

**Conclusion:**

Stress was a significant problem among study participants where a small proportion of study subjects presented with depression. Comprehensive counseling and guidance aligned with training on awareness creation were recommended.

## 1. Introduction

Stress is our body's response to pressures from a situation or life event called a “stressor” [1]. Academic stress is an unpleasant feeling of tension and anticipated frustration secondary to academic-related demands and expectations from significant others [2]. Depression is a common mental disorder characterized by a depressed mood, loss of pleasure, decreased energy, low self-worth, disturbed sleep, poor concentration, and feelings of guilt [3]. Mental health problems are significant causes of overall disease burden globally as it accounts for 21.2% years lived with disability worldwide. According to the World Health Organization (WHO) report in 2011, untreated mental health problems account for 13% of the total global burden of disease. By 2030, it is projected that mental health problems (particularly depression) will be the leading cause of mortality and morbidity globally. Recently, depression is estimated to affect 350 million people worldwide [4].

Report from the World Mental Health Survey conducted in 17 countries showed that on average 1 in 20 people has episodes of depression symptoms like a depressed mood, loss of interest and enjoyment, and increased fatigability in the previous year where a depressive episode is categorized as mild, moderate, or severe based on the number and severity of symptoms [5]. Over half of adults (51%) who felt stressed reported feeling depressed. Among those, 16% had self-harmed and 32% had suicidal thoughts and feelings [6].

Mental illness among university students will have numerous impacts on the individual, family, and community as undiagnosed or untreated mentally ill students are at high risk of dropouts from the academic program, losing their interest in studies, and depression, raising the unemployment rate that leads to extra burden on the families, society, and community as a whole. Evidence demonstrated that academic stress decreases the student's academic performance that hinders the ability to study efficiently and better time management [7, 8].

The main contributing factor of perceived stress was intrapersonal factors such as high self-expectation, new responsibility in life, lack of a friend, financial problem, and change in eating pattern while interpersonal stressors like high parental expectation and poor interpersonal relationships were the following one. In addition to these, environmental factors like poor dormitory facilities and change in a living environment were also stated as associated factors of stress [9].

Findings of Pondicherry University Indians revealed that 9.8% of students were suffering from high academic stress while 67.8% were victims of moderate academic stress. Findings from the public university of Sri Lanka among nursing students showed an alarming increase for the risk of mental health morbidity since study participants claimed mild to extremely severe symptoms of depression (51.1%) and stress (82.6%). A sense of financial insecurity and low social support were considered for high academic stress among the students coming from a lower socioeconomic background. Students' negative perceptions about the university academic environment and living arrangements were associated with depression. In addition to this, the students who felt academically stressed were significantly suffering from more depression [10, 11].

Evidence demonstrated at different universities of Islamabad revealed that academic stress decreases a student's academic performance hindering the ability to study efficiently and better time management. Academic stress is higher among juniors than seniors, as junior students are less experienced, have a low maturity level, and are more victimized by academic stress of university as compared to the seniors who are not only mature and experienced but also well adapted and well adjusted to academic stress [7].

The conducted surveys brought that health science streamed students had more stress compared to students studying in other streams mainly due to increased class workload and examinations. Students experiencing high levels of stress have difficulty learning, memorizing, and earning good grades as well as lead to poor physical, emotional, and mental health [12].

The presence and severity of depressive symptoms among health professionals' students at public universities in Riyadh, Saudi Arabia, were significantly associated with the female gender and the study year of participants [13].

The result of the public university of Ghana among midwives and nursing students brought that there is a high level of stress experienced by students due to academic and intrapersonal stressors [14]. Studies at the Tanta University of Egypt, Malaysian universities, Tarnow, Southern Poland, and the University of Baghdad reveal moderate to high levels of stress in which stressors were related to academics including clinical practice and social relationships [15–18].

Studies of King Saud University and Shahid Beheshti University indicate an alarming rate of depressive symptoms among health professional students of 44.2% and 20.6% from mild to moderate depression, respectively [13, 19]. Study results of university students in Nigeria showed that moderate to severe depression was found among students in which a lack of social support, sleeping problems, and poor academic performance were associated with depression [20].

The study conducted among health science students at Debrebran University explored that prevalence of the perceived stress level among health science students was 63.7%, in which gender, monthly income, social support, study year, and relationship with classmates and dormmates were associated factors among study participants [21].

Joining higher institutions is a transitional period from adolescence to adulthood for students. During this time, students independently perform a new level of responsibility such as planning their own daily tasks, managing their time, and budgeting their money. They start facing a variety of demands like difficulties in adjusting to the university culture and context, socioeconomic challenges, poor interpersonal relationships, intrapersonal problems, a heavily loaded curriculum, and limited institutional support. As a result, university students on average are highly vulnerable to stress and depression that leads them to poor performance, drop out of school, absenteeism, and decreased productivity thereby affecting their future carrier. Consequently, stress and depression have adverse effects on the wellbeing and academic performance of students if not identified early and managed effectively. In addition to this, there is a limited coping method and awareness about counseling and guidance service and negligible utilization of these services in the campus to decrease risk and improve students' psychological wellbeing [9].

Even though the different pieces of evidence suggest that there were a high magnitude and impact of a problem among study participants, few studies address mental health issues among health science students in higher institutions of Ethiopia. Therefore, assessing the current stress, depression, and associated factors among Arsi University students is highly important to recognize and take measures as early as possible.

## 2. Materials and Methods

### 2.1. Study Design, Period, and Setting

The institution-based cross-sectional study design was conducted in Arsi University from February 25 to April 15, 2019. Arsi University is located in the western Arsi zone Oromia regional state at a distance of 159 km away from Addis Ababa. The University is among the 3rd generation universities that newly started an education as a university in 2013 by the Ethiopian Ministry of Education (EME). Currently, 41 regular undergraduate departments are offering studies at six colleges and one school. The total regular students' population of this year is 5076. Among these, 2996 are males and 2080 females.

### 2.2. Sample Size Calculation and Sampling Procedure

The assumption made for sample size calculation was standard normal distribution with a 95% confidence interval, absolute precision or tolerable margin of error (*d* = 0.05), and prevalence of perceived stress among health science students at Debrebran University (63.7%) and considering the anticipated proportion of health science students who experience depression = 50% (*P*) since there was no similar study with this respect before. Then, the sample size is calculated for each dependent variable and took the maximum sample size (*n* = 384). A stratified random sampling method was used to get the required sample size in which strata was based on the department of study. Then, the numbers of study subjects in each stratum were determined by proportion to population size from each department. Then, eligible study subjects were further selected by simple random sampling.

### 2.3. Data Collection Instrument and Technique

Before the actual data collection, pretest on 5% (20 students) of the total sample size was carried out using a self-administered questionnaire on health science students at Madda Walabu University to ensure that the respondents are able to understand the questions and to check the wording and logic and skip the order of the questions in a sensible way to the respondents. Data was collected by using a self-administered questionnaire of the perceived stress scale (PSS-14) item and Beck depression inventory (BDI-II) 21 items while the students are in the classroom. Perceived stress is a measure of the degree in which a person assesses their life as the stressfulness of the situations in the past month of their life. The scale yielded a single score with high scores indicating higher levels of stress and lower levels indicating lower levels of stress. The BDI is scored by summing the ratings for the 21 items. Each item is rated on a 4-point scale ranging from 0 to 3; the maximum total score is 63. The questionnaires had four sections that contain sociodemographic data, perceived stress level, Beck depression inventory items, and questions to assess factors associated with stress and depression. Two instructors have collected data from study participants during the data collection period.

### 2.4. Operational Definitions

*Depression*: students' scores above 20 on the Beck depression inventory of 21 items.

*Minimal depression*: scores 1-13 on the Beck depression inventory scale of 21 items.

*Mild depression*: scores 14-19 on the Beck depression inventory scale of 21 items.

*Moderate depression*: individual scores 20-28 on the Beck depression inventory scale of 21 items.

*Severe depression*: individual scores 29-63 on the Beck depression inventory scale of 21 items.

*Stress*: students' scores above 28 cutoff values on the perceived stress scale.

*Perceived stress levels*: an individual's perceived response to interaction with his or her environment as measured by the perceived stress scale.

*No stress*: individual scores ranging from less than 14 on the PSS scores.

*Mild stress*: individual scores ranging from 15 to 28 on the PSS scores.

*Moderate stress*: individual scores ranging from 29 to 42 on the PSS scores.

*Severe stress*: individual scores ranging from 43 to 56 on the PSS scores.

*Health science students*: refers to nursing, midwifery, public health officer, pharmacy, medical laboratory, and anesthesia, according to this study.

### 2.5. Data Analysis and Interpretation

Collected data was each checked for completeness, and a code was given before data entry. Then, data were entered into EpiData version 4.4.2.1 and exported to Statistical Package for the Social Sciences (SPSS) version 21 software package for data analysis. Descriptive statistics were done to explore the distribution of sociodemographic characteristics, department of study, and study year characteristics of the study participants. Different frequency tables, graphs, and descriptive summaries were used to describe the study variables. Bivariate and multivariate logistic regression with an odds ratio (OD) of 95% confidence interval was used to identify the associated factors with dependent variables. Variables with a *P* value of less than 0.25 in the bivariable analysis were transformed into the multivariable analysis. In multivariable analysis, variables with a *P* value of less than 0.05 were considered statistically significant. The adjusted odds ratio (AOR) with the corresponding 95% confidence interval (CI) was used to show the strength of association.

## 3. Results

### 3.1. Sociodemographic Characteristics of the Study Participants among Health Science Students at Arsi University

All of the study participants complete the questionnaire, and this makes a 100% (384) response rate. From the study participants, 221 (57.6%) were male whereas 163 (42.4%) were females. All of the study subjects were between ages 18 and 30 with a mean age of 20.9 ± 1.83 standard deviation years. With regard to marital status, most of the study participants were single (365 (95.1)) while only 19 (4.5%) were married. Concerning enrollment to the field of study, 103 (26.8%), 74 (19.3%), 56 (14.6), 55 (14.3%), 52 (13.5), and 44 (11.5%) were from nursing, pharmacy, medical laboratory, public health officer, midwifery, and anesthesia departments, respectively. Among these, the majority of respondents were from second-year students (149 (38.8%)), followed by third-year students (114 (29.7%)). From those students who participated in the study, 269 (70.1%) were satisfied with their field of study, while the rest (115 (29.9%)) were unsatisfied with their field of study. The monthly mean income of respondents was 603.35 Ethiopian birr (±SD 494.7). More than half of respondents (229 (59.6%)) earn >500 birrs per month. Among the study respondents, 149 (38.8%) followed Orthodox while the rest 115 (29.9), 100 (26.0%), 18 (4.7%), and 2 (0.5%) were following Muslim, Protestant, others, and Catholic, respectively (Table 1).

### 3.2. Prevalence of Perceived Stress and Depression among Health Science Students at Arsi University

The overall prevalence of perceived stress among health science students was 63.5% in which the majority of students presented with a moderate stress level (235 (61.1%)) followed by a mild stress level, no stress, and severe stress (125 (32.6%), 15 (3.9%), and 9 (2.3%), respectively). The proportion level of perceived stress among study participants was relatively high among anesthesia students (70.5%) followed by pharmacy students (68.9%), whereas medical laboratory, midwifery, public health officer, and nursing constituted 67.9%, 63.5%, 58.2%, and 57.3% from highest to lowest proportion of perceived stress, respectively.

It is found that the prevalence of depression among study participants was 4.4. From this, almost two-thirds (72.9%) of study participants presented with minimal depression while 19.5% and 7.6% presented with mild depression and moderate depression levels, respectively. The proportion of depression in each department was 7.1% within the medical laboratory, 6.8% within anesthesia, 5.8% within midwifery, 3.9% within nursing, 3.6% among public health officer, and 1.4% among pharmacy (Table 2).

### 3.3. Factors Associated with Perceived Stress among Health Science Students at Arsi University

In bivariate analysis, age and study year from sociodemographic factors; the burden of study, the pressure to maintain a good grade, year of study, practical attachment, interpersonal relationship with a patient, their family, and medical personnel from academic-related stressors; changing environment and lack of dormitory safety from environment-related stressors; and high parental expectations and financial problem from psychosocial-related stressors fulfilled the minimum requirement (0.2 level of significance in this study). For further assessment, the data were entered into the multivariate logistic analysis. In the same manner, the result of multivariate analysis showed that there was a statistically significant association between perceived stress and students' age, study years, the pressure to maintain good grades, assigned to practical attachment, lack of dormitory safety, high parental expectation, and financial problem. The result of this study suggests that students of age ranging from 20 to 24 years were 0.56 less likely to be stressed compared to students of age less than 20 years. This showed that as the age of students increases from 20 to 24 years, perceived stress was decreased by half compared to students less than 20 years old. Second-year students were 0.357 less likely to be stressed compared to first-year students. Furthermore, third-year students were 0.290 less likely to present with perceived stress than first-year students.

As this finding shows, students pressured to maintain good grades were 1.903 times more likely to be stressed than students that are not pressured to maintain a good grade. Similarly, respondents assigned to practical attachment were 2.302 times more likely to be stressed compared to those students not assigned to practical attachment. Besides, this present study also pinpointed that inadequate dormitory safety was significantly associated with perceived stress levels in which students who lack dormitory safety were 1.698 more likely to be stressed compared to their counterparts. Moreover, the study also revealed that students who have high parental expectations were 2.011 times more likely to be stressed compared to those who had no high parental expectations. Finally, students who have financial constraints were 1.780 more likely to be stressed compared to those who had no financial problems (Table 3).

### 3.4. Factors Associated with Depression among Health Science Students at Arsi University

From the listed possible factors, poor academic performance and thinking about future career prospects showed statistical significance with depression on the bivariate analysis (0.2 level of significance in this study). But multivariate analysis showed that students thinking about future career prospects were 8.41 times more likely to be depressed compared to those who did not think about their future career prospects (Table 4).

## 4. Discussion

The cross-sectional study design was undertaken among 384 Arsi University health science students to assess perceived stress, depression, and associated factors. The result of the study indicated that there is an alarm in mental health morbidity among study participants, thus needing immediate attention as it may contribute to psychological, physical, and behavioral problems.

The high prevalence of perceived stress in the current study was almost consistent with the result of a similar study done at Debrebran University [21]. But it is 17 times higher than another study finding among regular health science students at Debrebran governmental colleges [22].

In the same manner, it is also higher than the prevalence of perceived stress among health professionals' students at Makerere University, undergraduate male pharmacy and medical students of a tertiary educational institution in Saudi Arabia, and undergraduate female students of health and nonhealth cluster colleges of a public sector university in Dammam [23–25]. The possible reasons for this variability could be due to nonconducive learning and teaching setup, difficulty in adaptation to poor dormitory safety and insecurity, poor infrastructure in the compound, lack of recreational facilities, and absence of student guidance and counseling on the campus. Moreover, the discrepancy could be also due to the differences in the used study tools, study population, competitive and stressful academic life, and environmental factors such as separation from family members, poor accommodation, and any problem arising from the adolescent. However, the result of the present study was lower than the prevalence of another study report among health professional students in Bosnia and Herzegovina [26]. Possible explanations could be differences in sociodemographic characteristics of the study population, campus environment, and utilized sample size. Furthermore, the study also revealed possible associated factors such as age category, study year, the pressure to maintain a good grade, practical attachment, lack of dormitory safety, high parental expectation, and financial problem that contributes to perceived stress among study subjects. The finding of this study signifies that the stress level of study subjects was decreasing in the subsequent years of study. This might be attributed to a gradual adjustment of study subjects to well adapt to university culture and context; therefore, they are more able to cope with stress than students in the early years.

This finding is consistent with the results of studies of [21, 27]. But it is inconsistent with the finding of [14, 28, 29]. In addition to this, as the age of students increases, perceived stress decreases that could be due to the increased maturity level and being more experienced as age increases.

As a result of this finding, it showed that students pressured to maintain a good grade was 1.903 times more likely to be stressed than students that are not pressured to maintain good grades. This is supported by the study findings conducted at Ghana University among pharmacy students [30].

According to this study results, respondents assigned to practical attachment were 2.302 times more likely to be stressed compared to those students who were not assigned to practical attachment. This could be explained as lack of preparation for practical attachment, lack of proper supervision, poor interpersonal relationships with clinical staff, and pressure from instructors contributing to the high perceived stress of students. This is aligned with the finding of Tanta University, in Egypt by 2017 [17].

Besides, this present study also pinpointed that inadequate dormitory safety was significantly associated with the perceived stress level in which students with inadequate dormitory safety were 1.698 more likely to be stressed compared to their counterparts. This might be due to overcrowding in one dorm, inadequate water supply, dormitory poor sanitation, and insecurity. This is strengthened by the result finding at Jimma University [9]. Moreover, students who have financial constraints were 1.780 more likely to be stressed compared to those who had no financial problems. This indicates that although food and dormitory service is provided to the students by the university, students might be in need of money for entrainments, to print handouts, and to buy items needed for a hygienic purpose that make financial constraints a stressor among students.

Finally, gender, monthly income (pocket money), and satisfaction with the field of study are not significantly associated with perceived stress in this study whereas the significant association was found on a similar study conducted at Debrebran University and study in two Saudi universities, respectively [21, 31].

On the other hand, the overall prevalence of depression among study subjects was relatively very low compared to other similar studies done in Saudi Arabia and Malaysia [13, 32]. This might be attributed to the difference in the tool used and sociodemographic characteristics of the study population. The result of this study also explored that thinking about future career prospects was statistically significant with depression of study participants. Students thinking about future career prospects were 8.41 times more likely to be depressed compared to others. This might be explained by issues related to students tense about future stable job opportunities, job searching, and an accessible chance for career advancement. This study result is in agreement with a similar study done at Shahid Beheshti University [19].

According to the outcome of this study, gender, age category, year of study, and departments were not statistically significant with depression. This is aligned with the finding of a study in Malaysia [33] and inconsistent with the study result of Saudi Arabia [13]. This discrepancy could be due to sociocultural differences in study populations.

## 5. Conclusion

To sum up, stress and depression were common mental health problems among health science students that need early recognition and interventions. There was a high prevalence of perceived stress (63.5%) among health science students. But the relatively low prevalence of depression (4.4%) was identified among study subjects. Age category, study year, the pressure to maintain a good grade, practical attachment, inadequate dormitory safety, high parental expectations, and financial problems were identified as factors associated with perceived stress, whereas thinking about future career prospects was identified as a factor associated with depression.

## Figures and Tables

**Table 1:** Sociodemographic characteristics of the study participants among health science students at Arsi University in 2019 in Oromia, Ethiopia (*n* = 384).

Variables	Number	Percent (%)	Mean ± SD
Age			20.9 (±1.83)
Income			603.35 (±494.75)
Sex			
Male	221	57.6	
Female	163	42.4	
Marital status			
Single	365	95.1	
Married	19	4. 5	
Field of study			
Nursing	103	26.8	
Pharmacy	74	19.3	
Medical laboratory	56	14.6	
Public health officer	55	14.3	
Midwifery	52	13.5	
Anesthesia	44	11.5	
Educational study year level			
First year	109	28.4	
Second year	149	38.8	
Third year	114	29.7	
Fourth year	12	3.1	
Satisfaction with field of study			
Satisfied	269	70.1	
Unsatisfied	115	29.9	
Religion			
Orthodox	149	38.8	
Muslim	115	29.9	
Protestant	100	26	
Others	18	4.7	
Catholic	2	0.5	

**Table 2:** Prevalence of stress and depression among health science students of Arsi University in 2019 in Oromia, Ethiopia (*n* = 384).

Category	Number	Percent (%)	Mean ± SD
Stress			28.4 (±7.60)
No stress	15	3.9	
Mild stress	125	32.6	
Moderate stress	235	61.2	
Severe stress	9	2.3	
Depression			9.72 (±5.93)
Minimal depression	280	72.9	
Mild depression	75	19.5	
Moderate depression	29	7.6	
Severe depression	0	0.0	

**Table 3:** Factors associated with perceived stress among health science students of Arsi University in 2019 in Oromia, Ethiopia (*n* = 384).

Variables	*P* value	Adjusted odds ratio (AOR, 95% CI)
Age		
20-24 years	0.017	0.562 (0.351, 0.901)^∗∗^
>24 years	0.471	0.650 (0.201, 2.098)
Educational study year		
Second year	0.001	0.357 (0.198, 0.644)^∗∗^
Third year	0.000	0.290 (0.153, 0.548)^∗∗^
Fourth year	0.196	0.432 (0.121, 1.544)
Burden of study	0.566	1.178 (0.673, 2.064)
Pressure to maintain good grade	0.005	1.903 (1.218, 2.973)^∗∗^
Year of study (batch)	0.083	1.521 (0.946, 2.444)
Practical attachment	0.003	2.302 (1.328, 3.991)^∗∗^
Interpersonal relationship at practice	0.712	1.137 (0.575, 2.247)
Changing environment	0.083	1.460 (0.952, 2.239)
Inadequate dormitory safety	0.018	1.698 (1.094, 2.636)^∗∗^
High parental expectation	0.002	2.011 (1.299, 3.113)^∗∗^
Financial problem	0.009	1.780 (1.153, 2.749)^∗∗^

^∗∗^ *P* value < 0.5.

**Table 4:** Factors associated with depression among health science students of Arsi University in 2019 in Oromia, Ethiopia (*n* = 384).

Variables	*P* value	Adjusted odds ratio (AOR, 95% CI)
Poor academic performance	0.206	2.330 (0.628, 8.638)
Thinking about future career prospect	0.046	8.415 (1.039, 68.138)^∗∗^

^∗∗^ *P* value < 0.5.

## Data Availability

The datasets used and/or analyzed during the study were available from the corresponding author on reasonable request through email bokaabdisa@yahoo.com.

## Abbreviations

AAU: Addis Ababa University
BDI: Beck depression inventory
CI: Confidence interval
E.C.: Ethiopian calendar
EME: Ethiopian Ministry of Education
OD: Odds ratio
PSS: Perceived stress scale
RRC: Research review committee
SD: Standard deviation
SPSS: Statistical Package for the Social Sciences
WHO: World Health Organization.
